# Desorption of 1,3,5-Trichlorobenzene from Multi-Walled Carbon Nanotubes: Impact of Solution Chemistry and Surface Chemistry

**DOI:** 10.3390/nano3020289

**Published:** 2013-05-17

**Authors:** Xingmao Ma, Sheikh Uddin

**Affiliations:** Department of Civil and Environmental Engineering, Southern Illinois University Carbondale, Carbondale, IL 62901, USA; E-Mail: sheikh.uddin@siu.edu

**Keywords:** multi-walled carbon nanotubes, 1,3,5-trichlorobenzene, desorption, surface functionalization, solution chemistry

## Abstract

The strong affinity of carbon nanotubes (CNTs) to environmental contaminants has raised serious concern that CNTs may function as a carrier of environmental pollutants and lead to contamination in places where the environmental pollutants are not expected. However, this concern will not be realized until the contaminants are desorbed from CNTs. It is well recognized that the desorption of environmental pollutants from pre-laden CNTs varies with the environmental conditions, such as the solution pH and ionic strength. However, comprehensive investigation on the influence of solution chemistry on the desorption process has not been carried out, even though numerous investigations have been conducted to investigate the impact of solution chemistry on the adsorption of environmental pollutants on CNTs. The main objective of this study was to determine the influence of solution chemistry (e.g., pH, ionic strength) and surface functionalization on the desorption of preloaded 1,3,5-trichlorobenzene (1,3,5-TCB) from multi-walled carbon nanotubes (MWNTs). The results suggested that higher pH, ionic strength and natural organic matter in solution generally led to higher desorption of 1,3,5-TCB from MWNTs. However, the extent of change varied at different values of the tested parameters (e.g., pH < 7 *vs*. pH > 7). In addition, the impact of these parameters varied with MWNTs possessing different surface functional groups, suggesting that surface functionalization could considerably alter the environmental behaviors and impact of MWNTs.

## 1. Introduction

The unique mechanical, electrical and magnetic properties of multi-walled carbon nanotube (MWNTs) lend great potential to their applications in biomedical engineering, environmental engineering, energy storage and biosensors. The applications and the subsequent implications of MWNTs in environmental engineering derive primarily from their very large specific surface area (SSA) and consequently very large adsorption capacity and strong affinity to a large number of environmental pollutants. As a result, MWNT has been widely studied as a novel adsorbent for a wide variety of environmental pollutants. Earlier results suggested that both the unique structure and surface properties of MWNTs and the physicochemical properties of adsorbate affect the adsorption capacity and affinity of environmental pollutants to MWNTs [1,2,3].

Aromatic compounds, such as chlorophenols or chlorobenzenes, can form π-π interactions between the graphene sheet of CNTs and the benzene ring of aromatic compounds, due to the electronic polarizability of graphene surface on MWNT sidewalls. Compounds containing strong electronegative functional groups generally form stronger bonds with the MWNT surface, due to the electron donor-acceptor interactions.

After purification, MWNTs generally contain certain functional groups, such as the hydroxyl or carbonyl group, depending upon the purification process and synthesis procedure. Functional groups can also be purposely added by oxidation process [4] or removed by certain physical and chemical treatment (e.g., heat treatment) [5]. Well-controlled experiment can incorporate over 10% of oxides by weight on CNTs surface [6]. The presence of surface functional groups can affect the adsorption process in several ways. For example, the functional groups can significantly alter the overall magnitude of π-π interactions between the adsorbed molecules and graphene sheets by increasing strong medium-range interactions linking π-orbitals of the substituents [7]. Zhang and colleagues [8] reported that surface functional groups of CNTs improved their dispersion in aqueous solutions, but decreased adsorption capacities for the hydrophobic synthetic organic compounds (SOCs). The reduced adsorption was contributed to the formation of water clusters around the oxygen-containing functional groups. Cho *et al*. [6] also showed that surface oxides on CNTs create polar regions that reduce the surface area available for naphthalene sorption. Gotovac *et al*. [9] studied the influences of different functional groups and diameters of CNTs on the adsorption of poly-aromatic hydrocarbons (PAHs) and found that acid functionalized CNTs had higher absorbability for the poly-aromatic molecules. MWNT surface can also be modified with other chemicals. For example, NH_3_-treated MWNTs showed higher adsorption capacity for chlorophenols than as-grown or HNO_3_-treated MWNTs. Liao and colleagues [3] suggested that better π-π dispersion and hydrophobic interaction are the main driving forces for the enhanced adsorption of NH_3_-treated MWNTs for chlorophenols.

In addition to the surface chemistry, solution chemistry also plays a critical role in the adsorption and desorption process. Solution chemistry can alter the speciation of both adsorbates and the functional groups on the adsorbent. Chen and Zhu [10] studied the effects of pH, ionic strength (IS) and the divalent metal ions, as well as the dissolved humic acid (HA) on the adsorption of nonionic aromatic compounds to single-walled carbon nanotubes (SWNTs). They found that divalent ion demonstrated little impact on the adsorption of nonionic aromatic compounds, while the presence of HA sharply reduced adsorption. Increasing pH induced deprotonation of the acidic functional groups (–COOH, –OH) on CNT surface and enhanced the π-electron-donor ability of the graphene surface and, thus, strengthened π-π electron-donor-acceptor (EDA) interactions of the aromatics. For ionizable organic compounds, the difference in solution pH could change the chemical speciation and, therefore, changing their adsorption characteristics. Higher pH usually leads to increased ionization, solubility and hydrophilicity. Changes in pH were also found to alter the adsorption of organic compounds to activated carbon and black carbon by modulating the surface characteristics of the adsorbents and the electronic properties of the adsorbate molecules [11,12,13]. Ionic solutes can also affect the adsorption of organic compounds. Chen and colleagues [14] demonstrated that copper ion decreased the adsorption of organic chemical to wood charcoal, because of the development of hydration shell, while silver ion increased the adsorption of organic chemicals, due to the reduced hydrophilicity of local region surrounding the silver ion.

The greatest effect, however, may come from natural organic matters (NOMs) in the environment. A main function of NOM is that it facilitates the suspension of CNTs in solution [15,16,17,18]. Surface coating of NOM was postulated as the predominant mechanism for enhanced dispersion [19]. The break of CNT bundles provide more adsorption sites and, thus, improve adsorption [20,21]. On the other hand, the adsorption of NOM on CNT surface occupies some adsorption sites and reduces the available spaces for organic compounds. The net effect of NOM hinges on the relative importance of these competing processes and the interactions between NOM organic compounds and the CNT surface.

Despite the significant insights obtained on the physical and chemical mechanisms concerning the adsorption of environmental pollutants to CNTs, current understanding on the desorption process of environmental pollutants from CNTs is still limited. According to a few available studies in the literature, the desorption of polycyclic aromatic hydrocarbons from MWNT bundles did not exhibit any hysteresis, due to the inability of tubular MWNTs to form porous structures [22]. However, a later study showed that desorption hysteresis was observed for a different compound, bisphenol A [23], suggesting that the molecular structure of adsorbates affect the desorption behavior of organic compounds. Previous research also indicated that desorption of organic compounds appeared to follow the two-stage desorption patterns from soils, and the desorption kinetics is dependent on the adsorption energy between the adsorbate and adsorbent, hence the initial concentration of adsorbate on CNTs [24]. In spite of these new insights, the desorption process is still understudied, and new knowledge concerning the impact of environmental conditions and surface properties of CNTs on the desorption process is needed.

With the rapid buildup of MWNTs in the environment and their strong affinity and large adsorption capacity of some environmental pollutants, concerns have emerged that MWNTs may function as a contaminant carrier in the environment and pose unexpected hazardous risks in places where these contaminants are not expected. Hyung [16] reported that NOMs and CNTs form stable complexes in aqueous solution, which can be transported to different locations with water, and similar interactions can occur between typical environmental pollutants and CNTs. To address this concern, it is important to understand the mechanisms of desorption of environmental pollutants from MWNTs under different environmental conditions. Limited previous research suggested that CNT surface properties, such as the presence of amorphous carbon, affect the desorption characteristics of chlorinated compounds [25,26,27]; yet, detailed investigation is still lacking. The objective of this study was to elucidate how the surface chemistry, such as the functionalization of MWNT surface, and the solution chemistry affected the desorption of pre-laden 1,3,5-trichlorobenzene from MWNTs. 1,3,5-TCB was chosen, because it is a common environmental pollutant and it is not readily biodegradable. The adsorption and desorption are important processes controlling their environmental fate and transport. 1,3,5-TCB is also very toxic. Some recent reports also showed that 1,3,5-TCB is a suspected endocrine disruptor and has been included in the European Union (EU) List of Substances with Suspected Endocrine Effects [28].

## 2. Results and Discussion

### 2.1. Effect of Ionic Strength

Overall, less than 10% of 1,3,5-TCB were desorbed from MWNTs into the aqueous phase in the batch reactors used in this study under different treatment conditions (e.g., different ionic strength (IS), pH or NOMs). Nevertheless, the desorption of 1,3,5-TCB revealed interesting trends under the impact of different solution chemistry parameters. A one-way ANOVA analysis indicated that the desorption rate was significantly affected by the tested parameters (*p* < 0.05 for all treatments).

**Figure 1:**
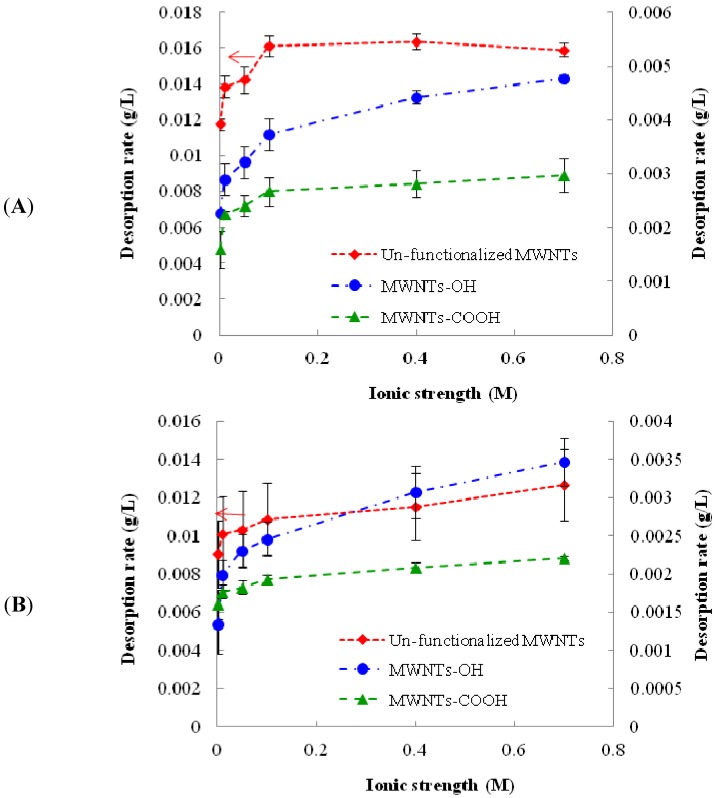
Change of the desorption rate of pre-loaded 1,3,5-TCB from multi-walled carbon nanotube (MWNTs) with different solution ionic strength and composition. (**A**) solutions containing CaCl_2_; (**B**) solutions containing NaCl. The reported values are the average of three or four replicates. Error bars stand for standard deviation.

In this study, a broad range of ionic strength (IS) was chosen to mimic the IS in freshwater and seawater. The results suggested that desorption of pre-loaded 1,3,5-TCB increased with increasing IS for all MWNTs, irrespective of the nature of functionality on CNT surface. However, the impact appeared to be stronger for un-functionalized MWNTs than functionalized MWNTs, especially at lower ionic strength (IS < 0.1 M). The observation may be explained by the fact that unfunctionalized MWNTs still contain some functional groups, even though they are not as concentrated as functionalized MWNTs. Unfuntionalized CNTs also contain higher amorphous carbon and catalysts; yet, their role on the impact of ionic strength on 1,3,5-TCB desorption is not clear. The relatively large error bars for un-functionalized MWNTs were also attributed to the heterogeneity of the un-functionalized MWNT surface. The ion composition of IS showed a difference in term of the IS impact on the desorption process, with the divalent Ca^2+^ demonstrating a stronger effect than the monovalent Na^+^. The result of the IS effect is shown in Figure 1.

Increasing ion concentrations in the solution could result in the compression of the double layers surrounding MWNTs and, consequently, led to the aggregation of MWNTs. The aggregates of MWNTs could be more compact (squeezing-out) and unfavorable for 1,3,5-TCB adsorption [29]. Increased IS also strengthened the electronic shielding effect on negatively charged MWNT surface and weakened the electrostatic forces, which contributed to the adsorption of 1,3,5-TCB to MWNTs. An earlier study suggested that the electron donor-acceptor interaction between –Cl on 1,3,5-TCB and the benzene rings on the CNT surface is the primary molecular force governing the adsorption of 1,3,5-TCN on CNT surface, even though other molecular forces, including the Van der Waals forces, also contribute to the adsorption [30,31]. With increasing ions in the solution, more ions would likely accumulate on the CNT surface, especially the polar regions, and reduce the adsorption of 1,3,5-TCB. The ions could also form a cation-π interaction, which has been shown to inhibit the electrostatic interactions, favoring the desorption of 1,3,5-TCB from CNT surface [31].

In this study, desorption rate was generally higher for un-functionalized MWNTs than functionalized MWNTs. The presence of functional group makes the MWNTs more hydrophilic and more dispersed in water, resulting in a more accessible surface for 1,3,5-TCB [32]. The ion composition effect is understandable in that the bivalent Ca^2+^ ion can cause a larger electronic screening effect compared with the monovalent Na^+^ ion and, hence, greater suppression on 1,3,5-TCB adsorption on carbon nanotubes [33]. The cation-bridging mechanism may also help interpret the observed phenomenon, because metal ions have been reported to bridge with the functional groups on CNTs by compressing the double layer and, thus, weaken the repulsion between CNTs, which eventually leads them to form aggregate, resulting in less adsorption and more desorption [34,35,36].

### 2.2. Effect of pH

**Figure 2:**
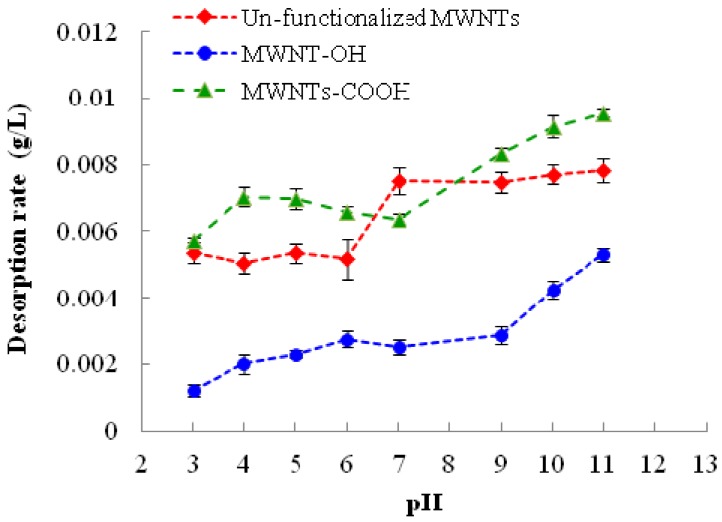
Change of the desorption rate of pre-loaded 1,3,5-TCB from MWNTs with solution pH. The reported values are the average of three or four replicates. Error bars represent standard deviation.

The effect of solution pH on the desorption of 1,3,5-TCB from MWNTs are shown in Figure 2. It is apparent that higher pH favored the desorption of 1,3,5-TCB for all MWNTs. The desorption of 1,3,5-TCB displayed somewhat similar patterns for the two functionalized MWNTs, but differed from un-functionalized MWNTs. In the acidic range, the effect of pH was insignificant for un-functionalized MWNTs and MWNTs-COOH, but lead to a gradual increase of 1,3,5-TCB desorption from MWNTs-OH. At a higher pH range, the desorption of 1,3,5-TCB was stable for un-functionalized MWNTs. However, the desorption of 1,3,5-TCB continued to increase with the pH at a basic range for both functionalized MWNTs.

It is widely recognized that pH shift could cause the protonation-deprotonation transition of the functional groups on MWNTs and the ionizable compounds. It has been reported that the adsorption of ionizable compounds on CNTs alters with pH, due to the chemical speciation [15,37,38]. 1,3,5-TCB is non-ionizable and will maintain neutrality throughout the pH range. Therefore, the observed effect should be attributed to the effect of pH on the deprotonation of functional groups on the CNT surface. The deprotonation of the hydroxyl and carboxyl functional groups on functionalized MWNTs at higher pH would generally make the sites more hydrophilic and encourage the formation of water clusters and discourage the adsorption of 1,3,5-TCB to CNTs, because hydrophobic adsorption was inhibited.

According to Dubinin [39], the water molecules adsorbed to the oxygen groups on carbon surface become secondary adsorption sites, which retain other water molecules by means of hydrogen bonding. Formation of water clusters around oxygen groups may affect the adsorption of 1,3,5-TCB by hindering the access of 1,3,5-TCB to the MWNT surface. The formation of water clusters on carboxyl groups has been suspected to block adsorption on the carboxyl sites [40] and result in more desorption.

One observation, which is worth mentioning, is that the desorption of 1,3,5-TCB from MWNTs-OH increased slowly from pH 3 to 9 and then jumped at pH > 9. The result may stem from the fact that the pK_a_ values of most R-OH compounds are greater than 11. At pH > 9, about two units around the pK_a_, deprotonation increased dramatically, compared with pH < 9, resulting in rapid building up of water clusters and more desorption of 1,3,5-TCB. In contrast, most R-COOH compounds have a much smaller pK_a_, and most of them are expected to fully deprotonate at neutral pH. Therefore, the rapid increase of desorption from MWNTs-COOH after pH 7 may not be explained by the protonation-deprotonation theory. It is likely that after pH 7, the hydroxide ion in solution had a stronger interaction with the electrophilic carbon on the CNT surface and weakened the EDA relationship between MWNT surface and 1,3,5-TCB by lowering the charge density on the oxygen molecule. The reason for the gradual drop of desorption from pH 4 to pH 7 for MWNTs-COOH is not clear. Unfunctionalized MWNTs typically contain some functional groups, even though they are more diverse and generally to a lower extent than functionalized MWNTs. The general similarity of the pH effect on all MWNTs investigated in this study may reflect this fact. However, unfunctionalized MWNTs typically possess amorphous carbon and defects on their surface, and it is believed that these features play a pronounced role in the adsorption and desorption process; their effects on the desorption of 1,3,5-TCB and other organic contaminants need further investigation.

### 2.3. Effect of Natural Organic Matter (NOM)

**Figure 3:**
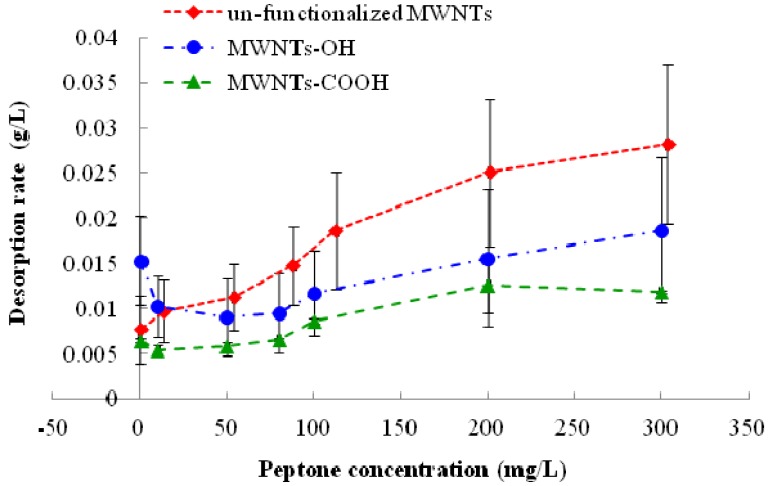
Change of the desorption rate of pre-loaded 1,3,5-TCB from MWNTs with concentrations of peptone in solution. The reported values are the average of three or four replicates. Error bars stand for standard deviation.

The desorption of 1,3,5-TCB under the influence of different concentrations of NOMs is shown in Figure 3. Increases of NOM in solution led to higher desorption for all types of MWNTs. It is generally accepted that NOM plays a crucial role in the adsorption to and desorption from CNTs for all organic compounds. The roles NOMs play are often contradictory in terms of their effect on the adsorption and desorption process. On the one hand, NOMs could facilitate the disperse of CNT bundles in water and make more adsorption sites available for adsorbates, thus increasing the adsorption [41]. On the other hand, NOM would compete with organic compounds for the same adsorption sites or physically block some available adsorption sites for organic compounds, resulting in reduced adsorption. Even though NOMs adsorbed on the CNT surface can function as a new adsorbent, their affinity with organic compounds appears to be smaller than the affinity between CNTs and organic compounds [20]. Both of these competing effects are expected to occur during the adsorption and desorption process. With higher dispersion, more MWNT surfaces are exposed to water, which would enhance the desorption of 1,3,5-TCB to the liquid. When the sites become exposed, NOMs might “push away” some of the 1,3,5-TCBs and occupy these sites. However, the similar effect could also apply to 1,3,5-TCB; that higher dispersion efficiency would provide more available sites for 1,3,5-TCB. In this study, the increase of NOM concentration generally led to higher desorption, suggesting that this effect is more pronounced for NOM than 1,3,5-TCB. One exception is that the desorption dropped for MWNT-OHs when peptone concentration increased from 0 to 50 mg/L and then rose again with higher peptone concentration. It could be postulated that MWNTs-OH are more “soluble” than other MWNTs and a small fraction of NOM is needed to further disperse them. However, relatively low concentrations of NOM could not occupy all newly opened sites, leaving more space for the adsorption of 1,3,5-TCB. With the increase of NOM in solution, these sites became unavailable to 1,3,5-TCB, and the greater affinity of NOM with MWNTs-OH caused more desorption of 1,3,5-TCB. It is also recognized that NOM is a highly diverse group of large molecular materials and different types of NOMs demonstrate different characteristics of interactions with MWNTs. Peptone is considered as a “fresh” NOM and differs drastically from more mature NOMs, such as humic substances. How different properties of NOMs (e.g., age and degree of aromaticity) affect their interactions with MWNTs and environmental pollutants need more investigation.

Based on our studies, it is clear that both the solution chemistry and surface properties exert important impacts on the desorption of 1,3,5-TCB from MWNTs. In the natural environment, the solution chemistry is defined by a combination of individual factors, including the ones examined in this investigation. It is essential in future studies to evaluate how those different parameters synergistically affect the desorption of organic compounds from MWNTs. With the continued development in CNT synthesis, more diverse CNTs with unique surface properties will be developed. Future studies should also be extended to evaluate the impacts of other surface functional groups on the adsorption and desorption process.

This study provides important insights on the potential environmental implications of the rapid development of carbon-based nanotechnology for the environment. Even though the potential toxicity of CNT itself has been examined extensively, the environmental impact of CNTs resulted from their unique characteristics, such as their huge adsorption capacity for environmental pollutants, has not been fully characterized. Our results suggest that the secondary effect of CNTs vary at different environmental conditions (e.g., freshwater ecosystem *vs*. estuarine), and the synthesis and modification of surface properties alter the adsorption to and desorption from CNTs for environmental pollutants. Therefore, the potential environmental impact of CNTs needs to be further examined in specific environments. There have been studies to investigate the complex interactions of CNTs and different environmental parameters. What those interactions mean for the adsorption and desorption of environmental pollutants onto CNTs is not clear, but should be explored. The results also suggested that the environmental impact of CNTs with different surface properties vary, and more intensive studies on their environmental consequences will contribute to the manufacturing of CNTs with less environmental impacts.

## 3. Experimental Section

### 3.1. Chemicals

#### 3.1.1. 1,3,5-Trichlorobenzene (1,3,5-TCB)

1,3,5-TCB was purchased from Sigma-Aldrich with >99% purity. Selected physicochemical properties of this compound are listed in Supplementary Table 1 (Table S1). The molecular structure of 1,3,5-TCB is shown in Supplementary Figure 1A (Figure S1A). Due to the low solubility of 1,3,5-TCB in water, 5000 mg/L of stock solution of 1,3,5-TCB in hexane (>99%) was first prepared; this stock solution was then diluted with deionized (DI) water to different concentrations for the experiments as described below.

#### 3.1.2. Peptone

Peptone is commonly used as a representative fresh NOM [42]. Peptone used in this study was also purchased from Sigma-Aldrich. The molecular structure of peptone is shown in Figure S1B.

#### 3.1.3. Carbon Nanotubes

Non-functionalized multi-walled carbon nanotubes (MWNTs) and multi-walled carbon nanotubes functionalized with hydroxyl group (MWNT-OH) and carboxyl group (MWNT-COOH) were purchased from U.S. Research Nanomaterials, Inc. According to the manufacturer, the carbon nanotubes were synthesized by the chemical vapor deposition (CVD) method using cobalt, manganese, molybdenum or nickel as catalyst. All MWNTs contained more than 95% of CNTs by weight as determined by thermo gravimetric analysis (TGA). The carbon content was more than 97% for all samples. Other important properties of these MWNTs reported by the manufacturer are summarized in Supplementary Table 2 (Table S2).

### 3.2. Preloading MWNTs with 1,3,5-TCB

About 0.5–0.6 g of MWNTs was weighed into a 22 mL vial, and the vial was then filled with DI water to its capacity. The vial was spiked with 1,3,5-TCB stock solution (5000 mg/L) to result in a final liquid concentration of 50 mg/L. The volume of hexane in the mixture was kept below 0.1% (*v*:*v*) to minimize the co-solvent effect. The vial was then quickly sealed with an aluminum foil-lined Teflon screw cap and placed on a rotary tumbler for 7 days at room temperature (20–23 °C) to achieve equilibrium. The literature has suggested that 7 days are typically adequate for the adsorption and desorption of organic compounds from CNTs to reach equilibrium [24]. At the end of equilibrium, the vial was left on a bench for 1 day to allow MWNT aggregates to settle. The supernatant was transferred to a new vial with a pipette and centrifuged at 14,000 rpm for 10 min to further separate water and MWNTs, which might have been dispersed in the solution, due to tumbling. Our result suggested the fraction is minimum compared with the total MWNTs used for loading. About 10 mL of the supernatant was then filtered through 0.2 μm (polytetrafluoroethylene-PTFE) filter. The filtrate was collected and extracted with hexane in a 1:1 ratio (*v*:*v*). For the extraction, the hexane and water mixture was shaken vigorously several times and then was tumbled for 3 more days to enhance extraction. Our test with solutions containing a known amount of 1,3,5-TCB suggested that the extraction efficiency of hexane is more than 95%. Following the mixing and separation of the hexane and water phase, 1 mL of solution from the hexane layer was transferred into a Gas Chromatography (GC) vial, and the liquid was directly analyzed for 1,3,5-TCB with a Trace Ultra GC affiliated from an electron capture detector (ECD). ECD is highly sensitive for chlorinated compounds, and the detection limit is in the low µg/L level, significantly lower than the concentrations used in this study. The TCB concentration in the filtrate was determined through a four-point standard curve. The 1,3,5-TCB loaded on MWNTS was then calculated based on the following mass balance equation:

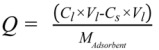
(1)
where *Q* is the solid concentration, in mg, of 1,3,5-TCB/g MWNTs; *C*_in_ is the concentration of 1,3,5-TCB in liquid (in mg/L) before equilibrium (50 mg/L); *V*_l_ is the volume of the vial (22 mL); *C*_e_ is the concentration of 1,3,5-TCB in filtrate (mg/L) determined through GC measurement; *M* is the mass of MWNTs (g).

The experiment was conducted similarly for both functionalized MWNTs to load 1,3,5-TCB on these CNTs. The results suggested that MWNTs-OH had greatest affinity for 1,3,5-TCB, followed by MWNTs-COOH and then unfunctionalized MWNTs. Through the adjustment of the amount of MWNTs used in the loading process, the actual concentrations of 1,3,5-TCB on MWNTs were controlled within the range of 2.004–2.346 mg TCB/g MWNTs for all MWNTs in different treatments. 

### 3.3. Desorption Experiment

The preloaded MWNTs slurry were used to investigate the desorption of 1,3,5-TCB at different environmental conditions. For the desorption study, a certain amount of the CNT slurry was added to a new vial. The vial was then filled with DI water to its capacity and sealed with an aluminum foil lined Teflon cap. The mixture was then tumbled for 7 days in a tumbler. The liquid and MWNTs were separated and 1,3,5-TCB in the liquid quantified, similarly as described above. The precise weight of MWNTs added to the vial was determined at the end of the desorption experiment by drying the residues at 103 °C for a day and calculating the difference between vials containing MWNTs and the blank vial, which was measured before the desorption experiment. The desorption rate defined as the ratio of the concentration of 1,3,5-TCB in water (mg/L) divided by the concentration on MWNTs (mg/g) after equilibrium was then calculated as:
Desorption rate, (*K*) = 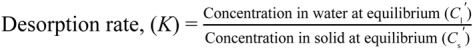
(2)
where *C*_l_*'* is the liquid concentration at equilibrium after the desorption study and was determined through the GC measurement. *C*_s_*'* is the solid concentration at equilibrium after desorption and was calculated through the following equation:

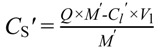
(3)
where *Q* is the solid concentration obtained from the adsorption study (mg/g), *M'* is the mass of preloaded MWNTs added to the vial in the desorption study and *V*_l_ is the volume of the vial (22 mL).

### 3.4. Solution Chemistry Parameters

Three factors, including the solution pH, ionic strength (IS) and NOM (represented by peptone) were evaluated for their influence on the desorption of pre-laden 1,3,5-TCB from MWNT surface. The pH evaluated ranged from 3 to 11. The IS investigated ranged from 0 (DI water) to 0.7 M (the typical IS of sea water). The IS solution was prepared with either NaCl or CaCl_2_ to investigate the ion composition effect. Peptone was used as a representative of fresh natural organic matter (NOM) in this study. A very wide range of peptone concentration (0.35–300 mg/L) was selected to represent the varying amount of NOMs in different natural environments. The weight effect of NOM was ignored, due to their relative insignificance, compared to the weight of MWNTs. Three or four replicates were prepared for each treatment.

## 4. Conclusions

In summary, the desorption of 1,3,5-TCB from MWNTs was affected by both the solution chemistry and the functional groups on the MWNT surface. Higher pH, ionic strength or NOMs in solution generally led to more desorption of 1,3,5-TCB from the MWNTs surface, even though the total 1,3,5-TCB desorbed from MWNTs represented only a small fraction of total 1,3,5-TCB in the systems. However, the dynamics could be drastically different in an open system as in the actual environment. Addition of oxygen-containing functional groups (–OH and –COOH) tended to reduce the desorption of 1,3,5-TCB and strongly affected the desorption patterns of 1,3,5-TCB. In addition to the ionic strength of a solution, the ion composition should also be an important consideration in the study of adsorption and desorption of organic compounds. Multivalent ions showed a stronger effect on the desorption of 1,3,5-TCB than monovalent ions. The results suggested that the desorption of environmental pollutants from MWNTs varies with the environmental conditions, and the secondary risks associated with MWNTs of different surface properties need to be evaluated separately.

## Supplementary Materials

Supplementary File 1
