# Supplementary material for: Desorption of 1,3,5-Trichlorobenzene from Multi-Walled Carbon Nanotubes: Impact of Solution Chemistry and Surface Chemistry
Source: Nanomaterials (Basel). 2013 May 17;3(2):289–302. doi: 10.3390/nano3020289 (PMC5327886; doi:10.3390/nano3020289)
Supplement: Supplementary File 1 [file nanomaterials-03-00289-s001.pdf]

Supplementary Information

Table S1. Physicochemical properties of 1,3,5-TCB *.

Compounds	Molecular weight (g/mol)	Solubility (mg/L)	Octanol-water partitioning coefficient (log k_{ow})	Henry's law constant (atm-m^3/mol)
1,3,5-TCB	181.45	6.01	4.19	0.00189

* The physical properties of this compound were obtained from the SRC PhysProp Database http://www.syrres.com/esc/physdemo.htm.

Table S2. Physicochemical properties of MWNTs *.

CNTs type	Non-Functionalized MWNTs	MWNT-OH	MWNT-COOH
Functional Groups	None	5.58 wt.% of –OH (XPS & Titration)	3.86 wt.% of –COOH (XPS & Titration)
Outside diameter	5–15 nm (from HRTEM, Raman)	<7 nm (from HRTEM, Raman)	<7 nm (from HRTEM, Raman)
Inside diameter	3–5 nm	2–5 nm	2–5 nm
Length	~50 μm (TEM)	0.5–2 μm (TEM)	10–30 μm (TEM)
SSA	> 233 m^2/g (BET)	500 m^2/g (BET)	500 m^2/g (BET)
Tap Density	0.27 g/cm^3	0.27 g/cm^3	0.27 g/cm^3
True density	~2.1 g/cm^3	~2.1 g/cm^3	~2.1 g/cm^3

* Source: http://www.us-nano.com/nanotubes#a4.

Figure S1. (A) molecular structure of 1,3,5-trichlorobenzene; (B) molecular structure of peptone.

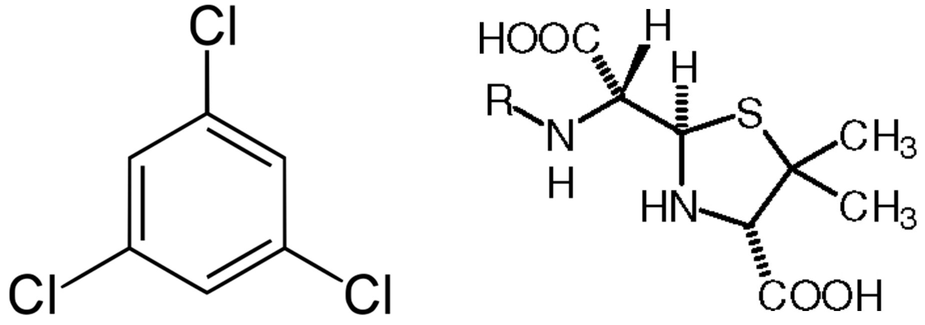

(**A**) (**B**)

© 2013 by the authors; licensee MDPI, Basel, Switzerland. This article is an open access article distributed under the terms and conditions of the Creative Commons Attribution license (http://creativecommons.org/licenses/by/3.0/).
